# Effects of tissue decalcification on the quantification of breast cancer biomarkers by digital image analysis

**DOI:** 10.1186/s13000-014-0213-9

**Published:** 2014-11-25

**Authors:** Arkadiusz Gertych, Sonia Mohan, Shawn Maclary, Sambit Mohanty, Kolja Wawrowsky, James Mirocha, Bonnie Balzer, Beatrice S Knudsen

**Affiliations:** Department of Pathology and Laboratory Medicine, Cedars-Sinai Medical Center, Los Angeles, CA USA; Departments of Surgery, Cedars Sinai Medical Center, 116N Robertson Blvd. Suite 903, Los Angeles, CA 90048 USA; Departments of Biomedical Sciences, Cedars Sinai Medical Center, 116N Robertson Blvd. Suite 500, Los Angeles, CA 90048 USA; Department of Biostatistics, Cedars-Sinai Medical Center, Los Angeles, CA USA; Current address: Division of Pathology and Laboratory Medicine, Loma Linda, CA USA; Current address: Surgical Pathology, Super Religare Laboratories and Fortis Hospital, Delhi, India

**Keywords:** Tissue decalcification, Breast cancer biomarkers, Image analysis, Quantification

## Abstract

**Background:**

Recent technical advances in digital image capture and analysis greatly improve the measurement of protein expression in tissues. Breast cancer biomarkers provide a unique opportunity to utilize digital image analysis to evaluate sources of variability that are caused by the tissue preparation, in particular the decalcification treatment associated with the analysis of bone metastatic breast cancer, and to develop methods for comparison of digital data and categorical scores rendered by pathologists.

**Methods:**

Tissues were prospectively decalcified for up to 24 hours and stained by immunohistochemistry (IHC) for ER, PR, Ki-67 and p53. HER2 positive breast cancer sections were retrieved from the pathology archives, and annotated with the categorical HER2 expression scores from the pathology reports. Digital images were captured with Leica and Aperio slide scanners. The conversion of the digital to categorical scores was accomplished with a Gaussian mixture model and tested for accuracy by comparison to clinical scores.

**Results:**

We observe significant effects of the decalcification treatment on common breast cancer biomarkers that are used in the clinic. ER, PR and p53 staining intensities decreased 15 – 20%, whereas Ki-67 decreased > 90% during the first 6 hrs of treatment and stabilized thereafter. In comparison with the Aperio images, pixel intensities generated by the Leica system are lower. A novel statistical model for conversion of digital to categorical scores provides a systematic approach for conversion of nuclear and membrane stains and demonstrated a high concordance with clinical scores.

**Conclusion:**

Digital image analysis greatly improves the quantification of protein expression in human tissues. Decalcification affects the accuracy of immunohistochemical staining results and cannot be reversed by image analysis. Measurement data obtained on a continuous scoring scale can be converted to categorical scores for comparison with categorical dataset that are generated by pathologists.

**Virtual Slides:**

The virtual slide(s) for this article can be found here: http://www.diagnosticpathology.diagnomx.eu/vs/13000_2014_213

**Electronic supplementary material:**

The online version of this article (doi:10.1186/s13000-014-0213-9) contains supplementary material, which is available to authorized users.

## Background

Digital image analysis is used increasingly to quantify protein expression in human tissues. A computer-assisted approach has advantages compared to the conventional, visual assessment of staining intensities in terms of quantification, dynamic range, reproducibility and sensitivity [1,2]. However, like any method it is affected by technical variability that is incompletely understood. Here we systematically examine sources of technical error that originate from the imaging system and tissue preparation. We also demonstrate a statistical approach to convert the staining intensity that is measured by the instrument into a categorical score that is familiar to pathologists and researchers reporting protein expression in tissues.

Immunohistochemistry (IHC) has advantages and disadvantages over fluorescent detection of protein expression. IHC generates a permanent staining record, depicts the tissue architecture for accurate diagnosis and permits amplification of the signal using a number of post-amplification reagents (polymers and tyramide) [3,4]. However, the disadvantages of IHC are its lack of linear signal amplification, the difficulties to utilize multiple antibodies simultaneously and the imprecision of colorimetric measurements. Further disadvantages include the intra- and inter-observer inconsistencies of reporting staining intensities. Therefore, there is a need to seek computer assistance to improve the accuracy and reproducibility of IHC- based measurements and to establish a systematic approach for converting digital into visual, categorical scales that are used by pathologists.

There are multiple systems available for generation of digital images and for their analyses. The Leica SCN400 and Aperio ScanScope AT Turbo instruments (Leica Biosystems, Buffalo Grove, IL) generate high-resolution whole slide images at a rapid rate and according to manufacturers’ label both devices provide image quality that is suitable for diagnostic pathology and pathology research. Since digital images may slightly differ from direct visualization of slides through the light microscope, pathologists dictate the ultimate acceptance and utilization of the slide scanning instruments in clinical practice, education and research.

There are human weaknesses in the visual assessment of stained slides that can be overcome by analytical software. Pathologists train for many years in pattern recognition and no single software package today parallels the diagnostic skills that acquired through this training. While human observers poorly distinguish shades of coloration at low staining intensities or estimate percentages of regions with specific features (low versus high staining intensities, or different grades) [5], machine vision techniques can overcome these weaknesses. The computer-assisted reproducibility of immunohistochemical scoring constitutes a critical factor in the development of biomarkers for clinical applications. There exist several commercial [6–8] and open source software packages [9–12] to quantify breast cancer biomarkers by image analysis. Existing systems can measure the immunoreaction product (brown diaminobenzidine (DAB) precipitate) that correlates with the abundance of the HER2/ErbB2 protein and discriminate between cells that are negative, weak and dark in terms of brown color. The FDA recently approved software that is incorporated into the Aperio slide scanner. The software contains an algorithm to covert digital to categorical scores of HER2 expression in breast cancer [8] and demonstrates that changing continuous to categorical formats of immunohistochemical data is feasible.

Utilizing a commercial system for IHC analyses (Leica Biosystems Buffalo Grove, IL) we quantified the effect of the tissue preparation on the measurement of biomarkers that are used in the clinic for prognosis and treatment of patients with breast cancer. We probed the effects of a tissue decalcification agent on nuclear expressions of ER, PR, p53 and Ki-67. Optical properties of the Leica system were qualitatively compared to an FDA cleared hardware-software system Aperio (Leica Biosystems, Buffalo Grove, IL). We also applied a statistical model to covert digital DAB intensity scores to categorical scores. The combined approach for quantification of nuclear and membranous immunostains should allow for an easier adaptation and a more common utilization of digital pathology platforms for biomarker development.

## Methods

### Tissue and decalcification process

Cancer tissues from 9 serial breast cancer cases with a large tumor volume were collected prospectively. The tissue samples were obtained from the excess that was left over after sections were submitted for routine pathologic evaluation. The analyses of immunohistochemal markers in these cases was considered exempt by the Cedars-Sinai Medical Center’s Institutional Review Board (IRB) (not subject research), since the research only included remnant tissues and medical records were not accessed. The only information collected from pathology reports was the tumor stage. The data were attached to the specimen immediately when the report became available and the connection to the report permanently destroyed right away, rendering future case identification impossible. Tumor samples were fixed in 10% neutral buffered formalin for longer than 6 hours, but less than 48 hours as per College of American Pathologist’s guidelines [13–15]. Subsequently, 2.5 × 2.0 × 0.3 cm tissue pieces were placed in cassettes, washed in water and decalcified using a hydrochloric acid-based decalcification solution (Decal State, Decal Chemical Corp., Tallman, NY). The decalcification times were 0, 1, 6 or 24 hours. Cassettes were washed extensively after decalcification and stored in 70% ethanol until processing.

Leica PELORIS premium tissue processor (Leica Biosystems, Buffalo Grove, IL) was used for tissue processing, that involved: 10% neutral buffered formalin for 20 min at 45°C; 70% ethyl alcohol for 20 min at 45°C; 90% ethyl alcohol for 20 min at 45°C; 100% ethyl alcohol for 20 min at 45°C; 100% ethyl alcohol for 20 min at 45°C; 100% ethyl alcohol for 20 min at 45°C; 100% ethyl alcohol for 20 min at 45°C; xylene for 30 min at 45°C; xylene for 30 min at 45°C; xylene for 60 min at 45°C; paraffin wax for 40 min at 65°C; paraffin wax for 40 min at 65°C; paraffin wax for 60 min at 65°C [16].

### Immunohistochemistry

Immunohistochemical staining with antibodies (all prediluted and purchased from Ventana Medical Systems, Tucson, AZ) for estrogen receptor (ER, mouse monoclonal clone SP1), progesterone receptor (PR, mouse monoclonal clone 1E2) human epidermal growth factor receptor-2 (HER2, rabbit monoclonal clone 4B5), cell proliferation marker Ki-67 (rabbit monoclonal clone 30–9), and p53 protein (mouse monoclonal clone D07) were performed on 4-μm thick tissue sections on the Ventana autostainer (Ventana Medical Systems, Tucson, AZ). Reagents and conditions, such as antigen retrieval and antibody incubations were all predefined by Ventana [17]. Bound secondary antibodies were visualized with 3,3-diaminobenzidine (DAB) chromogen substrate. After immunostaining, the slides were counterstained with haematoxylin.

### Cases selection, digital image acquisition, histopathological evaluation and image annotation

All cases expressed ER, PR and Ki-67. Four cases expressed p53 and only one case was positive for HER2. Only cases that were positive before decalcification treatment were selected for image analysis. To obtain an adequate cohort of HER2 positive cases, slides were retrieved from the pathology archives under an IRB approved protocol. These HER2+ cases were annotated with HER2 scores from the pathology report: 3 cases with HER2 = 0, 4 cases with HER2 = 1+, 4 cases with HER2 = 2+, and 4 cases with HER2 = 3+. A representative slide was selected by a pathologist (SM) for image analysis. 480 digital images were captured at × 20 magnification from 95 slides on the Leica SCN400 whole slide scanner (Additional file 1: Table S1). All images were saved as 24bit RGB color images in (*.svs) Leica platform-specific format and stored on a Digital Image Hub (DIH) (Leica Biosystems, Buffalo Grove, IL) for quantification. Using a self fabricated image annotation tool, a pathologist outlined up to 5 randomly selected tumor regions with approximately 2000 cells on each image for quantification (Additional file 1: Table S1).

### Qualitative evaluation of image capture in Leica and Aperio instruments

Before testing the accuracy of the conversion, we evaluated the effects of the instrumentation on the staining intensity (Additional file 2). To compare the basic image capture properties of the Leica SCN400 scanner and the Aperio ScanScope AT Turbo, one HER2+ and one ER + slide were randomly selected and digitized at 20 × magnification on both instruments. The normalized histograms from identical rectangular tissue regions in the three basic color channels were extracted for comparison. Monochromatic images of the same tissue area (one from SCN400 scanner, and one from Aperio ScanScope AT Turbo) were co-registered to measure local intensity correspondence. A graph showing correlation of output intensities was also formed.

### Image analysis and statistical evaluation

The Leica Tissue IA software package [18] was used for analysis. First, DAB images were deconvoluted [19] and then thresholded at t_0_ level light transmission to remove background and negative (with no DAB staining) pixels. t_0_ was set using a global histogram thresholding method [20]. For tissue images with nuclear ER, PR, p53 and Ki-67 staining, a nuclear segmentation procedure returned a nuclear mask for analysis. If the average DAB signal was above t_0_, the nucleus was considered negative. Otherwise it was positive. The percentage of Ki-67 positive nuclei was reported with its mean and standard deviation. For ER, PR and p53 stain, the average staining intensity in a delineated region was calculated. The average intensities at different decalcification times were normalized to the zero time point and plotted as a percentage of staining prior to decalcification. To evaluate differences between any two time points, distributions of the average intensities and percentages of positive cells were compared across all times for each marker. The distributions were approximately normal at each time except p53, and thus differences in means across the times were assessed by 1-way analysis of variance (ANOVA) models. For each ANOVA model, the significance was confirmed by Welch’s ANOVA on the ranks. Following a significant model, pairwise time comparisons were made using Tukey studentized range tests, which control the type 1 error rate. For p53 the Kruskal-Wallis test was used rather than ANOVA and pairwise time comparisons were made using the Wilcoxon rank sum test, with a Bonferroni-adjusted significance level of 0.05/6 = 0.0083 to evaluate differences between any two time points.

### Conversion of linear to categorical scoring

Four digitized ER + slides and four HER2+ slides graded as 0, 1+, 2+ and 3+ were selected. From each slide a square area (Field Of View, FOV_ER_ and FOV_HER2_) containing approximately 10,000 cancer cells with heterogeneous staining was selected, subjected to DAB color deconvolution and thresholding (described above). The intensity values of positively stained cells were collected and modeled as a mixture of Gaussian components. Since the clinical HER2 grading involves three categories (weak, moderate and strong staining), we fit a model of three Gaussian components and applied the Expectation-Maximization (EM) algorithm to model the three probability density functions. Each category was assumed to have a normal distribution, which was defined by a mean and a standard deviation *N* (*μ*, *σ*). *μ* and *σ* were determined by the expectation–maximization (EM) algorithm as those of maximum likelihood in the Gaussian mixture model. Initialization of EM was performed by the *k*-means algorithm. The mean and standard deviation (*μ*_1–3_, *σ*_1–3_) was calculated for each FOV and used to derive the Gaussian distributions. The thresholds (t_1_ and t_2_) for the staining categories are at the 2 points of intersection of the Gaussian distributions and were projected onto an intensity axis (abscissa) to define thresholds of DAB intensity categories. The derived ER and HER2 thresholds were applied to all images in the study and the fractions (range 0 – 1) of stained cells categories: weak, moderate and strongly were determined in each FOV. Fractions were multiplied by the category rank, providing values between 0 and 3. These values from HER2 stained slides were compared against pathologist grading and Aperio scoring.

## Results

Bone is a common site of metastatic breast cancer. In order to treat patients with bone metastasis, ER, PR and HER2 receptor status play important roles in treatment decisions and drug selections. Since bone biopsies are decalcified prior to immunohistochemical staining to prepare the bone for the generation of micro thin sections, the validity of biomarker measurements is endangered by the decalcification treatment. Here we utilize image analysis to determine the effects of decalcification, evaluate the measurement error that is caused by the instrumentation and develop a statistical approach to convert digital into categorical scores, which are routinely used by pathologists to communicate the immunohistochmical results to oncologists.

### Effects of decalcification on the quantification of nuclear breast cancer biomarkers in tissues

The advantages of a computer assisted approach over visual assessment in the quantification of immunohistochemistry are the consistency of the measurement, which eliminates intra-observer and inter-observer variability, the improved accuracy, in particular in the low intensity range and the accuracy in the enumeration of percentages of nuclear or cell surface signals. A typical workflow for quantification of nuclear or membranous protein expression in slides stained with DAB involves the following steps: (a) numerical separation of the DAB image from the haematoxylin image, (b) segmentation of nuclei based on the haematoxylin image or of cell membranes based on the DAB image, (c) determination of thresholds to quantify the percentage of cells with different levels of DAB positivity, and (d) enumeration of cells within levels of signal intensities defined according to (c).

**Figure 1:**
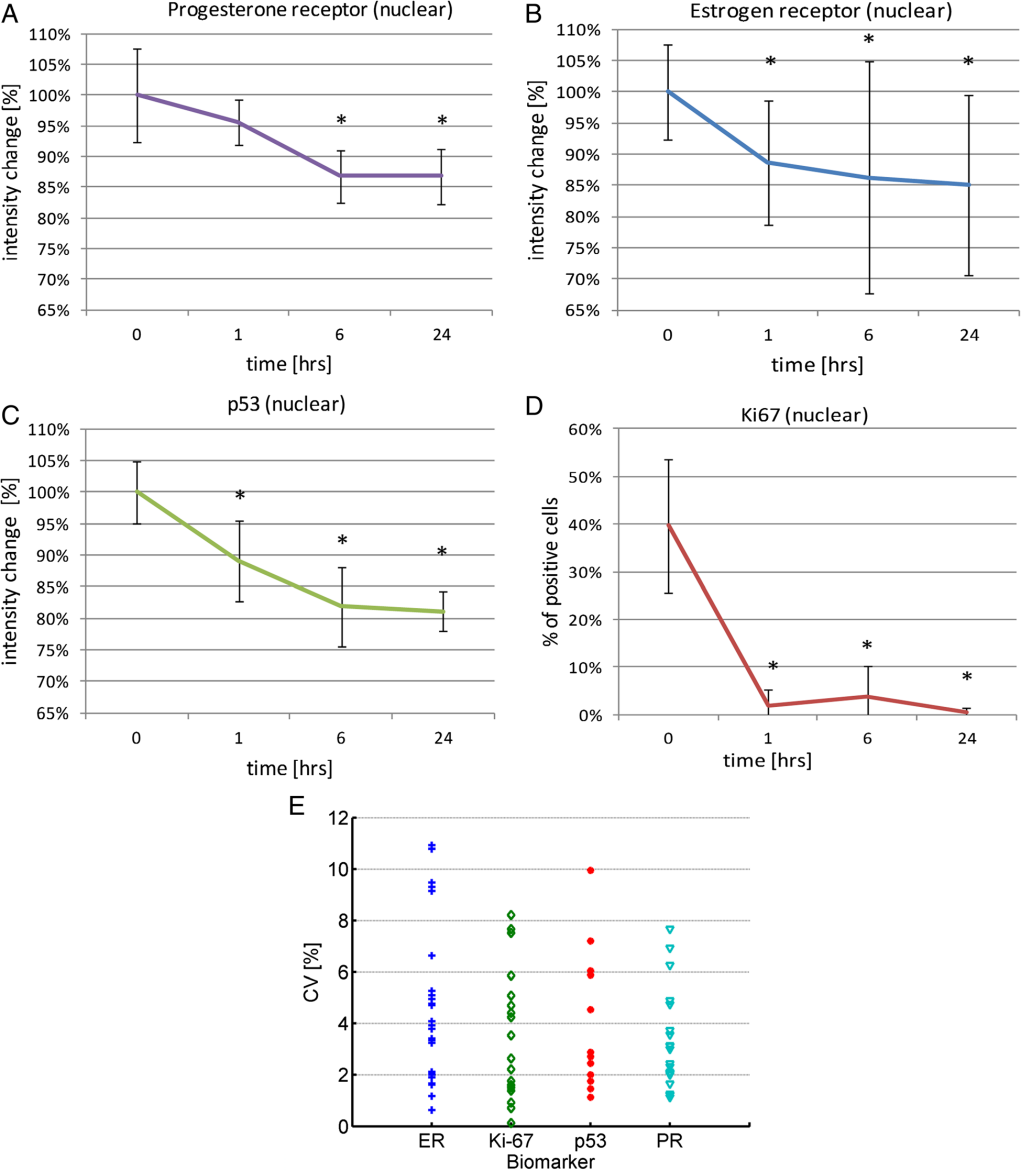
**Quantification of breast cancer biomarkers in tissues treated by decalcification: The study involved 9 cases and 5 fields per slide were measured at each time point.** **A - C)** The average staining intensities at each time point across all slides are plotted on the Y-axis for the estrogen receptor **(A)**, progesterone receptor **(B)** and p53 **(C)** and the % Ki-67 positive cells are platted in **D**. The X-axis indicates the time interval of the decalcification treatment. *p < 0.005 versus baseline. **E)** The %CV across 5 fields in each slide is shown for 29 slides stained for ER, 20 stained for PR, 12 stained for p53 and 18 stained for Ki-67.

To determine the effects of the decalcification time, tissue images were analyzed with image analysis software (Leica Tissue IA, Leica Microsystems, Buffalo Grove, IL) [18]. A progressive decline of staining intensity with time of decalcification was observed for all markers. The average loss for p53 was 20%, while ER and PR staining was reduced by 15% (Figure 1A-C). A significant decline (p < 0.005) occurred after the first hour of treatment. Longer treatment times of 6 and 24 hours did not cause further losses, demonstrated by insignificant differences in staining intensities between the 6 and 24 hours time points (p > 0.05). The greatest effect of the decalcification treatment was observed for Ki-67 immunoreactivity, which reports cell proliferation. Ki-67 staining decreased from an average of 35% to below the limit of detection after decalcification for 1 hr (p < 0.005) (Figure 1D).

To determine the heterogeneity of expression of each protein within the cancer, we calculated the % CV across the 5 areas that we measured in each slide. The intra-tumor heterogeneity ranged between 0.2 and 11% (Figure 1E). In no case did decalcification increase the staining intensity.

Overall, the effects of decalcification were significantly greater for Ki-67 than for ER, PR and p53. The decrease in staining intensity occurred rapidly, with a significant drop after treatment for 1 hour and stabilized between 6 and 24 hours of treatment.

### Conversion of linear staining intensity to categorical scoring

While ER, PR and Ki-67 are quantified routinely by image analysis during the clinical workup of breast cancers and are reported as the percentage of positive cells in the clinical pathology report, the clinical assessment of HER2 expression is based on a categorical 0, 1+, 2+ and 3+ scale. In general, pathologists use categorical scores to compare IHC staining intensities across cases and categorical scores are also used in most research projects that are designed to assess the role of IHC markers in diagnostic questions and in therapy and disease outcomes prediction. In order to compare studies that utilize image analysis to studies published by pathologists, the digital score of the computer image must be converted to a categorical score. Except for HER2, the methodology to accomplish this conversion in a standardized fashion is not available.

**Figure 2:**
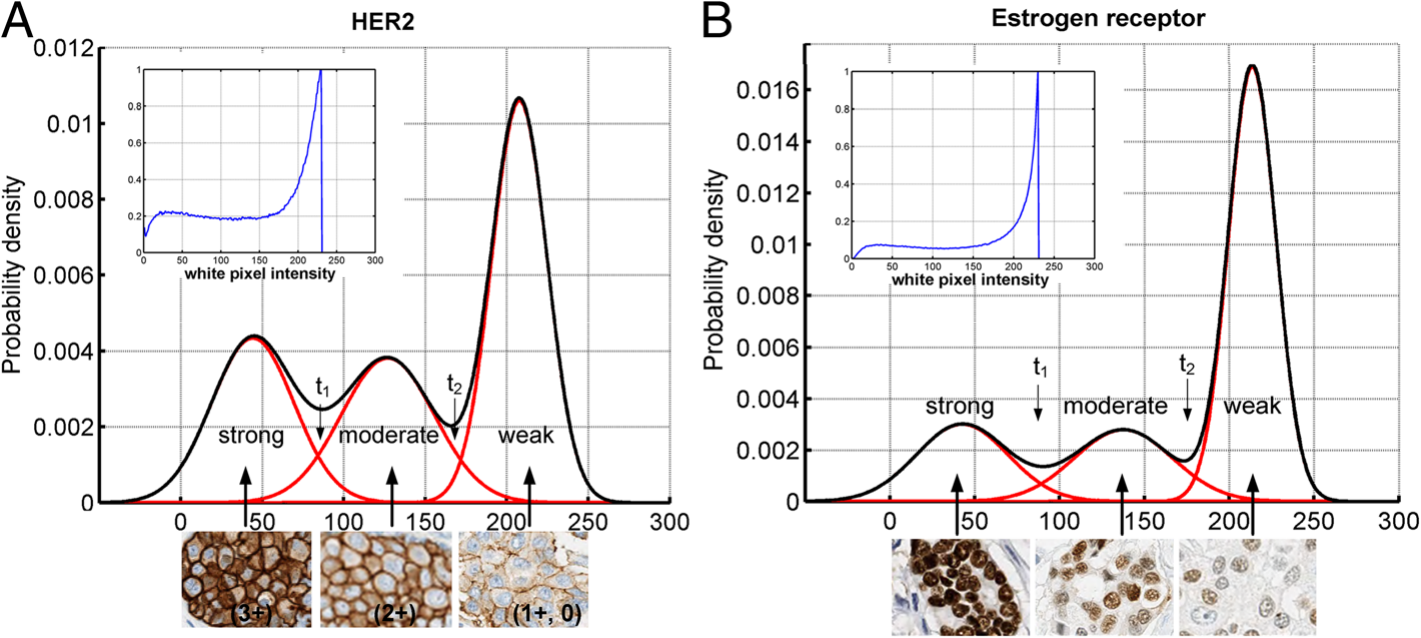
**Conversion of linear to categorical scoring.** The intensity histogram (panels inside **A** and **B**) obtained from a whole slide image was modeled using the Gaussian mixture. The Expectation-Maximization (EM) algorithm was applied to model three Gaussian components with the means and standard deviations for strong, moderate and weak intensity ranges. The white pixel intensity is plotted on the X axis with high values corresponding to less coloration. The probability density (Y axis) represents the frequency of pixels. A background cutoff was chosen arbitrarily at 230 white pixel intensity in DAB_HER2_ and DAB_ER_ images. Pixel intensities indicated by thresholds t_1_ and t_2_ separate 3+ from 2+ or 2+ from 1+/0 respectively.

To develop a conversion approach from digital to categorical scoring, approximately 10000 cells in multiple tumor areas with heterogeneous staining were extracted from 4 digitized slides stained either for HER2 or ER. Fields of view, FOV_HER2_ and FOV_ER_, were deconvoluted into separate images for DAB and hematoxylin. A threshold to eliminate the background was set at the level of t_0_ = 230. Pixel intensities lower than t_0_, which are indicative of positive staining were statistically modeled by the Gaussian mixture model and classified as strong, moderate or weak. The output of the model is shown in Figure 2 and consists of 3 distributions that intersect at t_1_ and t_2_, which represent the thresholds that separate strong from moderate, and moderate from weak staining categories. These thresholds are subsequently applied to categorize staining intensities of nuclei or cell membranes in the entire cohort. For HER2 immunohistochemical stains, the algorithm separated strong staining (3+) with values between 0 and 85, moderate staining (2+) with values from 86 to 180 and weak staining (0, 1+) 181 to 230 (Figure 2). For ER staining, cutoffs were < 83 for strong staining (3+), < 169 for moderate (2+) and between 170 and 230 for weak (0, 1+) staining.

Before testing the accuracy of the conversion, we evaluated the effects of the instrumentation on the staining intensity. Slides that were stained with the HER2 or ER antibodies were scanned on the Aperio and Leica slide scanners. Image intensity range 0–255 was divided into three zones: strong, moderate, weak and negative with background determined by thresholds from Figure 2A, and areas under the histogram (Additional file 3: Figure S1A and B) for each zone in red, blue, green and monochromatic channels zone were calculated (Additional file 1: Table S2). A comparison revealed larger areas under the curve that corresponded to pixel numbers in weak, moderate and strong staining intensity categories in the image that was acquired with the Leica scanner. On the contrary, Aperio image had substantially greater areas in the negative and background pixel ranges, indicating a rightward shift of the Aperio RGB histograms. In addition, at all white pixel intensities, greater white pixel numbers indicative of lesser color pixels were measured with the Aperio instrument for both HER2 and ER IHC stains (Additional file 3: Figure S1C and D). Altogether, these data demonstrate that the images captured with the Leica instrument are darker, compared to those from the Aperio instrument.

**Figure 3:**
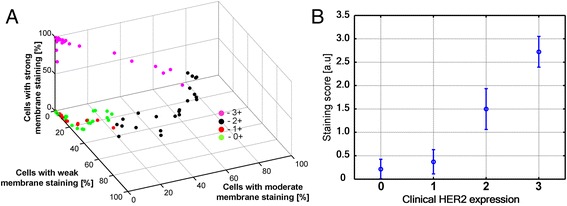
**Visualization of staining categories of Her2 membrane expression. A**) Each point represents one region from a slide. Data of weak, moderate and strong positive cells and of negative cells were collected. Three categories (1, 2 and 3) of positive cells were defined based on t_1_ and t_2_ thresholds in Figure 2. The percentages of cells in weak, moderate and strong staining categories are plotted on X, Y and Z axes. Note clusters of HER2 grade 0 and 1+ (green and red dots), HER2 grade 2+ (black) and 3+ (pink). **B**) Relationship between staining score (mean +/− std) and clinical HER2 expression. Staining scores were calculated by summing up the percentages of cells multiplied by 1, 2 and 3.

To test the accuracy of the conversion approach from digital to categorical scoring, we used clinical cases of HER2 positive and negative breast cancers from the pathology archives (n = 15). Breast cancer cases routinely undergo assessment for HER2 expression by pathologists and expression levels are communicated in the pathology report using a categorical scale of 0, 1+, 2+ and 3+. To compare the results obtained by our approach to the pathologist we calculated the average categorical score from image analysis results. Cancer cells in an area were assigned to weak, moderate or strong categories (Figure 3A) and the fraction of cells in each category (in a range of 0 to 1) was multiplied with the intensity score (1 – 3) to derive the average score of the image. As shown in Figure 3A cases clustered according to the assigned clinical grades. In addition, cases in each of the clinical score groups (0/1, 2, 3) fell into a digital score range that was not overlapping with the other groups, suggesting that the separation between intensity categories can be unequivocally accomplished (Figure 3B).

## Discussion

In clinical pathology practice, breast cancer biomarkers are used irrespective of the origin of the tissue and the process of sample preparation. Breast cancer frequently metastasizes to the bone. To examine bone biopsies, the tissue needs to be softened through decalcification. Immunohistochemistry for ER and PR is most commonly used to strengthen the diagnosis of breast cancer in bone biopsies. In addition, Ki-67 and p53 are used occasionally for prognostication and treatment guidance. The possibility that the decalcification process changes the intensity of the biomarker signals has not been addressed systematically. Therefore in this study, we examine the effects of the decalcification process on common breast cancer biomarkers. In order to determine the time dependence of the decalcification treatment, we use tissues form breast surgeries. We reasoned that decalcification effects will be similar in primary and bone metastatic cancer and that utilizing breast cancer in surgical resection provides an adequate starting point to test the consequences of decalcification on breast cancer biomarkers. To exclude the unlikely possibility that the presence of bone might influence the results, we added bone spicules to the decalcification treatment, but did not observe a change in IHC results (data not shown).

For all 4 markers, the signal decreased for up to 6 hours of treatment and then stabilized. It is encouraging that longer decalcification times (>6 hours), which are needed to soften the bone for the preparation of slides, do not cause excessive destruction of protein analytes. Since ER and PR are used to confirm a breast cancer diagnosis, the small decline in the signal (~15 - 20%) does not reduce the diagnostic utility. On the other hand, the massive loss of Ki-67 staining to the point where the signal is no longer detectable discourages the use of Ki-67 to assess cell proliferation in decalcified tissues, not only for breast cancer, but also for other cancer types in the bone.

Another study also reports a reduction in staining for Ki-67 after tissue decalcification. The European Bone Marrow Working Group assessed the effects of fixatives and decalcification protocols on immunohistochemical analysis of bone marrow biopsies. Amongst 6 markers that are routinely used in the workup of bone marrow biopsies for hematologic malignancies, Ki-67 and CD117 were the most problematic across the 19 hospitals that participated in the study [21]. Surprisingly, ER staining was less affected by the decalcification process than Ki-67, despite being characteristically sensitive to tissue photo-oxidation [22]. In addition to decalcification and photo-oxidation, the loss of immunoreactivity through slide storage is another worrisome possibility and reported to be more severe for PR and HER2 compared to ER [23]. Thus in addition to the breast cancer biomarker used in this study, each IHC marker needs be evaluated individually tested for its sensitivity to various pre-analytical conditions that might affect the accuracy of its measurement.

The measurement of protein expression by digital image analysis is advantageous, because it is more accurate, reproducible and sensitive at low staining intensities [5,7,8]. Several instruments are available for the acquisition of digital images, however comparing acquisition parameters between instruments is usually not possible, because investigators normally only have access to one instrument. We took advantage of the available Leica and Aperio instruments at our institution to compare the quality of their image acquisition. Both instruments generate high quality digital images, however, the Leica images are darker. The Leica instrument captures a larger number of color pixels in all 3 RGB channels and the numbers of white pixels are proportionally smaller. The correlation coefficient for the white pixel intensities comparing Leica and Aperio is >0.9 and the systematic error was consistent across pixel intensities (Additional file 3: Figure S1C and D). The observed differences in images captured by Leica and Aperio instruments may arise from differences in the optics and in the power of the light source, which affect the amplitude, position and width of the color components. A halogen bulb is used in the Aperio ScanScope AT Turbo, while a semiconductor-based light emitting diode device is used as the light source in the Leica SCN400 instrument [personal communication with Leica]. Images from the Aperio possess fewer blue pixels than those from the Leica instrument. Hence, the cell membranes in Leica images appear darker and the nuclear structure is finer. The closer proximity of histogram peaks in Aperio images to the limit of detection results in an overall weaker appearing stained slide. In addition the red histogram in the Leica image is shifted towards the high intensity range to a greater extent than the other color components, generating a “warmer” image. However, these results will unlikely affect study outcomes or conversion from digital to categorical scores.

Because of the superior performance of digital image analysis versus a human observer, the utilization of computer assisted image analysis is on the rise. However, the lack of a universal method to convert digital into categorical scores hinders the validation of digital image analysis by comparison with traditional image analysis systems. There are only a few examples of digital to categorical conversion methods. A conversion method was reported to classify pancreatic islets in diabetic rates as either normal or abnormal. The investigators employed a finite mixture mathematical model, which is similar to our algorithm and includes seven islet parameters from digital image analysis. The model possessed high accuracy for dividing islets into normal and abnormal categories [24]. Another example in the clinical molecular pathology laboratory is the conversion of digital to categorical scores that is included in the FDA approved analysis software of the Aperio slide scanner [25]. The software is limited to analysis of HER2 membrane staining and it is unclear whether it can be used for measurements other than HER2 stained slides. A weakness of these approaches, including our approach is the comparison of a pseudo-scientific categorical grading system, which has been clinically validated with a quantitative and more precise digital scale. A better method for comparison would be the analysis of samples by western blotting or molecular biology. However, this is not feasible for formalin fixed samples in this study.

To fill the need for more open source digital-to-categorical conversion systems for a broad range of applications, we developed an approach that is not restricted by the localization of the immunohistochemical signal. Our approach, which models strong, moderate and weak intensity intervals by fitting a Gaussian distribution curve within each intensity window, can be applied universally to IHC stained slides. The cutoffs for weak, moderate and strong staining are established with 4 images that span the entire immunohistochemical intensity spectrum of the project. The settings of cutoffs are statistically derived, independent of the observer, and require only a small set of training data. When the cutoffs were applied to a set of 87 regions from 15 HER2 slides, we calculated categorical scores that corresponded to those provided by the algorithm of the Aperio instrument. We would also like to point out that our amalgamated algorithm for conversion of linear to categorical scoring differs from that included in the Aperio software package which quantifies cells by membrane staining intensity and completeness. Although our solution is devoid of the latter constituent, it can still provide meaningful results.

Thus, by applying the algorithm we developed to convert digital to categorical scores, we can accomplish a valid comparison of digital and categorical datasets for IHC-stained slides for both membranous and nuclear markers.

## Conclusions

In summary, we use digital image analysis to accurately determine the effects of tissue decalcification on biomarkers that are in clinical use for the diagnosis and treatment of breast cancer. Our novel statistical method to generate data from digital images that can be compared to pathologists’ assessment of immunohistochemical stains provides the means for a standardized approach and a tool to compare digital data to those generated by pathologists.

## Additional files

Additional file 1: Table S1. Slide and region of interest numbers in the study. Regions of interests were outlined by a pathologist. Each region contained on average 2000 cells. **Table S2.** Normalized areas under the curve for image histograms acquired by Aperio and Leica instruments. Additional file 2:
**Methods.**
Additional file 3: Figure S1. Comparison of image capture properties between Leica and Aperio instruments. The same slide was imaged using Leica SCN400 and Aperio ScanScopeAT Turbo instruments. Panels A and B depict intensity histograms extracted from digital images obtained with Aperio and Leica respectively. Color lines indicate intensities in the red, green and blue channels as well as their grayscale transformation (black line). Inserts provide examples of slide contents from breast cancer samples stained with the HER2 antibody. Discrepancies in coloration in the immunohistochemistry images correspond to the differences in histogram shapes of all three channels. C) Relationships between white pixels acquired by Aperio and Leica slide scanners for a slide stained with HER2 antibody. The membrane staining was evaluated in the range of white pixels from 120 to 240 (See methods section). D) Same as C) but for a slide stained with ER antibody. A linear regression model was used to fit the data. **Figure S2.** Distribution of cells from all slides within three categories of HER2 staining intensity. Three staining categories were defined based on thresholds (t1 and t2) shown in Figure 1. Each slide received a pathological score of HER2 expression of 0, 1, 2 or 3. HER2 staining was quantified in five regions and the staining score was assigned to one of the three staining categories. Panel A shows the distribution of cells with negative or weak positivity in slides that were graded between 0 and 3 by a pathologist. Panel B, and C show the distribution of cells with moderate and strong positivity respectively. In total 87 regions from 15 slides were analyzed.
