# Supplementary material for: Effects of tissue decalcification on the quantification of breast cancer biomarkers by digital image analysis
Source: Diagn Pathol. 2014 Nov 25;9:213. doi: 10.1186/s13000-014-0213-9 (PMC4252006; doi:10.1186/s13000-014-0213-9)
Supplement: Additional file 1: Table S1. — Slide and region of interest numbers in the study. Regions of interests were outlined by a pathologist. Each region contained on average 2000 cells. Table S2. Normalized areas under the curve for image histograms acquired by Aperio and Leica instruments. [file 13000_2014_213_MOESM1_ESM.docx]

Gertych et al.

**Supplementary Table 1. Slide and region of interest numbers in the study.** Regions of interests were outlined by a pathologist. Each region contained on average 2000 cells.

| Marker | PR | | | | ER | | | | P53 | | | | Ki67 | | | | HE2 | Total |
| --- | --- | --- | --- | --- | --- | --- | --- | --- | --- | --- | --- | --- | --- | --- | --- | --- | --- | --- |
| Decal. Time [hrs] | 0 | 1 | 6 | 24 | 0 | 1 | 6 | 24 | 0 | 1 | 6 | 24 | 0 | 1 | 6 | 24 | NA | - |
| Slides | 6 | 1 | 5 | 8 | 8 | 3 | 8 | 10 | 4 | 2 | 2 | 4 | 6 | 3 | 4 | 5 | 15 | 95 |
| Regions | 30 | 5 | 25 | 40 | 40 | 15 | 40 | 50 | 19 | 10 | 10 | 20 | 29 | 15 | 20 | 25 | 87 | 480 |

**Supplementary Table 2.** Normalized areas under the curve for image histograms acquired by Aperio and Leica instruments.

| Device | Aperio TurboScan AT | | | | Leica SCN400F | | | |
| --- | --- | --- | --- | --- | --- | --- | --- | --- |
| Intensity rage  Color  channel | strong | moderate | weak | negative + background | strong | moderate | weak | negative + background |
| Red | 0.36 | 22.95 | 50.39 | 26.03 | 0.96 | 30.24 | 57.18 | 11.10 |
| Green | 1.49 | 30.47 | 46.77 | 21.17 | 3.00 | 39.23 | 52.28 | 5.34 |
| Blue | 4.53 | 33.07 | 42.09 | 20.12 | 5.90 | 39.01 | 50.18 | 4.66 |
| Gray | 1.09 | 29.28 | 47.67 | 21.9 | 2.48 | 36.98 | 53.93 | 6.48 |
