# Supplementary material for: Effects of tissue decalcification on the quantification of breast cancer biomarkers by digital image analysis
Source: Diagn Pathol. 2014 Nov 25;9:213. doi: 10.1186/s13000-014-0213-9 (PMC4252006; doi:10.1186/s13000-014-0213-9)
Supplement: Additional file 2: — Methods. [file 13000_2014_213_MOESM2_ESM.docx]

Gertych et al.

Supplementary methods

*Image capture properties of Aperio and Leica instruments*

State-of-the-art research settings are usually equipped with image analysis systems able to perform some but usually not all of the above steps. We utilized Tissue IA system (Leica, Buffalo Grove, IL) which can automatically perform steps (a) and (b), and (d) upon manual adjustment of thresholds in (c). However, the manual adjustment is subjective, and has poor reproducibility. In order to address the issues of subjective threshold selection, we implemented a systematic approach to convert the linear system of pixel intensities (0-255) to scoring categories defining weak, moderate and strong staining intensity, and applied it to quantify bioanalytes in nuclear and membranous compartments.

Properties of slide capture were qualitatively and quantitatively evaluated using I_HER2_L_, I_ER_L_, and I_HER2_A_ and I_ER_A_ images. First, normalized intensity histograms of I_HER2_A_ and I_HER2_L_ were juxtaposed (**Supplementary Figure 1A and B**). Next, I_HER2_A_ was aligned with I_HER2_L_ using the affine transform - in both images five control points were selected at the same locations to create a matrix with transform parameters. The transform was done using the image registration tool available in the Matlab software package (Mathworks Inc, Natick, MA). Co-registered images had exactly the same size and tissue content. Then, the co-registered images were converted to a gray scale according to the following formula: $Y=0.2989*R+0.587*G+0.1140*B$, where *Y* is the output pixel intensity and *R*, *G* and *B* are respective intensities in the red, green and blue channels. Lastly, the gray images were scanned from top-left to bottom-right corner by a 500x500 pixel window moving consecutively with a 100 pixel interval for every column and every row until the entire image was scanned. Mean values of image intensity from each window were recorded and then displayed as a function of white pixel intensity (**Supplementary Figure 1C**). The image registration, conversion to gray scale, and scanning were also applied to I_HER2_A_ and I_HER2_L_ images. Results are displayed in (**Supplementary Figure 1D**).
