# Supplementary material for: Effects of tissue decalcification on the quantification of breast cancer biomarkers by digital image analysis
Source: Diagn Pathol. 2014 Nov 25;9:213. doi: 10.1186/s13000-014-0213-9 (PMC4252006; doi:10.1186/s13000-014-0213-9)
Supplement: Additional file 3: Figure S1. — Comparison of image capture properties between Leica and Aperio instruments. The same slide was imaged using Leica SCN400 and Aperio ScanScopeAT Turbo instruments. Panels A and B depict intensity histograms extracted from digital images obtained with Aperio and Leica respectively. Color lines indicate intensities in the red, green and blue channels as well as their grayscale transformation (black line). Inserts provide examples of slide contents from breast cancer samples stained with the HER2 antibody. Discrepancies in coloration in the immunohistochemistry images correspond to the differences in histogram shapes of all three channels. C) Relationships between white pixels acquired by Aperio and Leica slide scanners for a slide stained with HER2 antibody. The membrane staining was evaluated in the range of white pixels from 120 to 240 (See methods section). D) Same as C) but for a slide stained with ER antibody. A linear regression model was used to fit the data. Figure S2. Distribution of cells from all slides within three categories of HER2 staining intensity. Three staining categories were defined based on thresholds (t1 and t2) shown in Figure 1. Each slide received a pathological score of HER2 expression of 0, 1, 2 or 3. HER2 staining was quantified in five regions and the staining score was assigned to one of the three staining categories. Panel A shows the distribution of cells with negative or weak positivity in slides that were graded between 0 and 3 by a pathologist. Panel B, and C show the distribution of cells with moderate and strong positivity respectively. In total 87 regions from 15 slides were analyzed. [file 13000_2014_213_MOESM3_ESM.docx]

Gertych et al.

**Supplementary Figure 1**

**
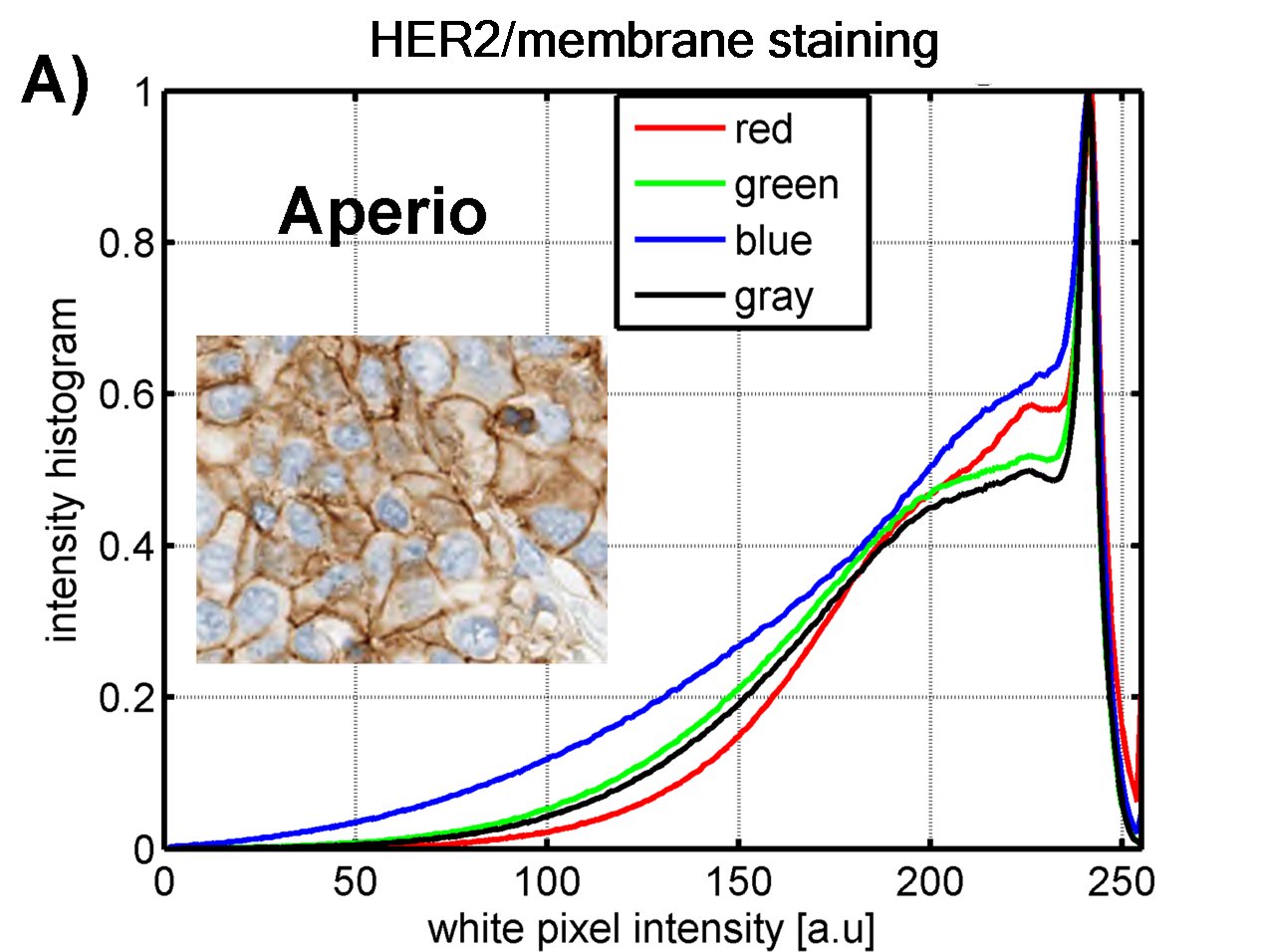
**
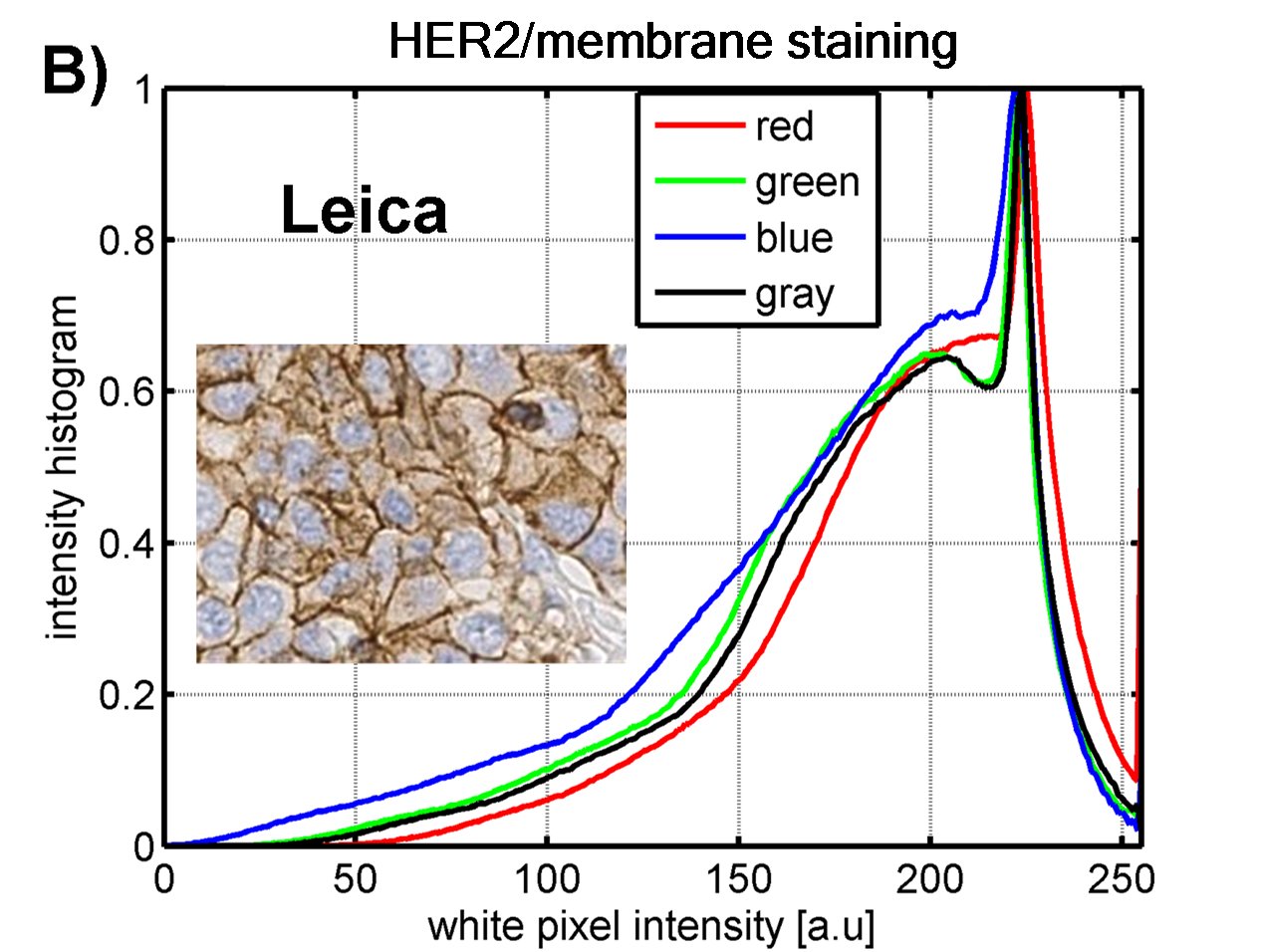


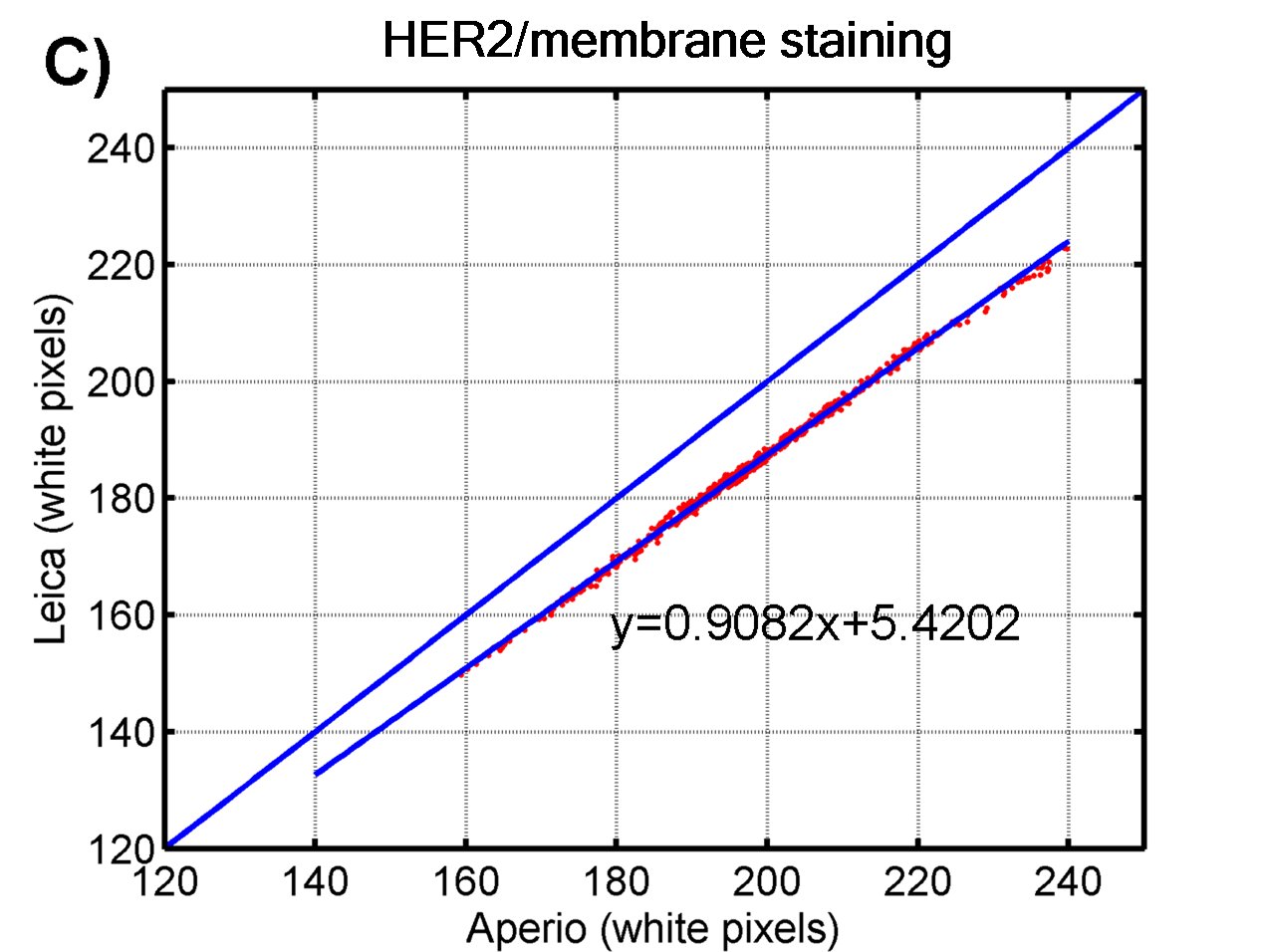

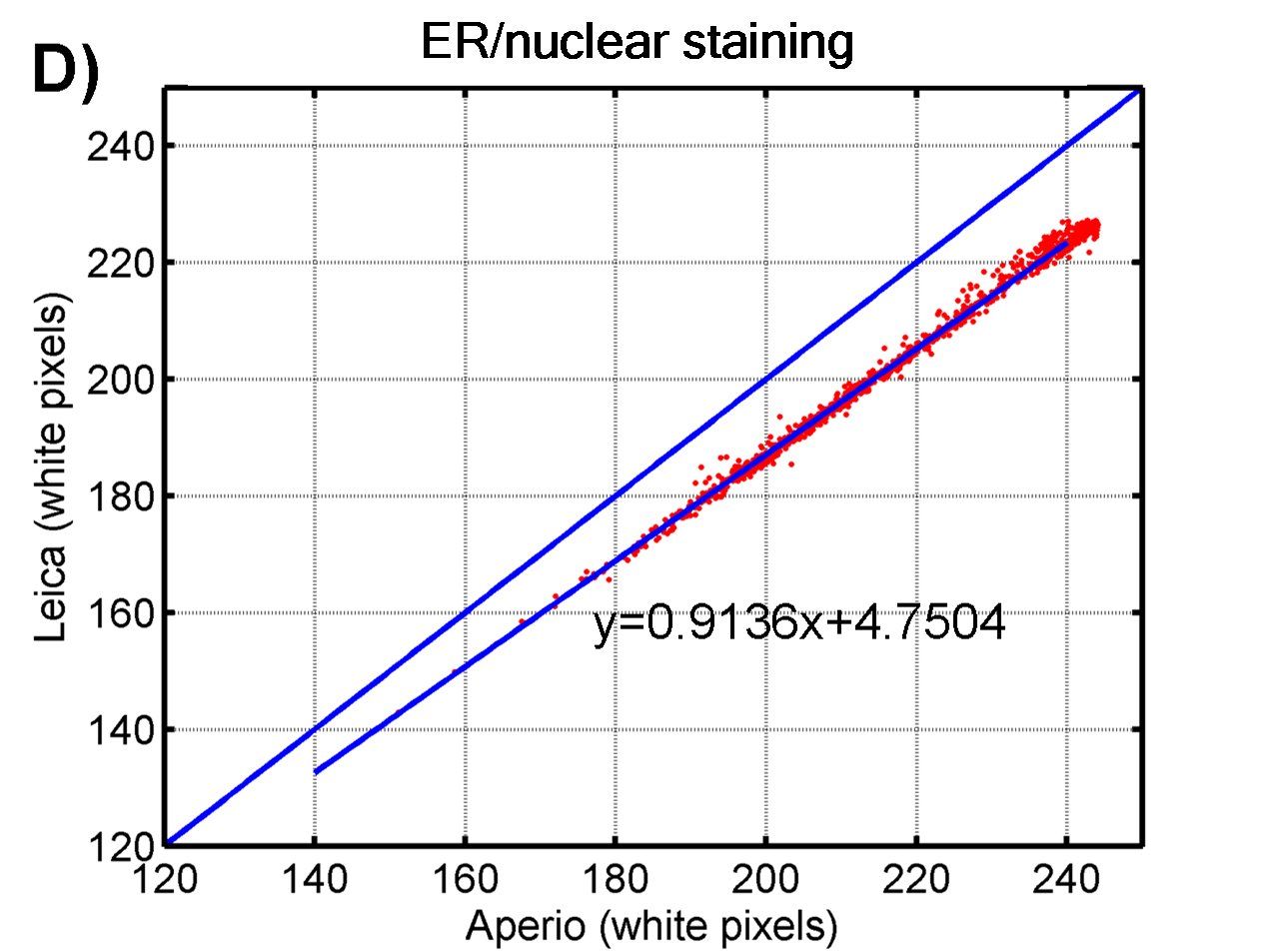


**Supplementary Figure 1. Comparison of image capture properties between Leica and Aperio instruments.** The same slide was imaged using Leica SCN400 and Aperio ScanScopeAT Turbo instruments. Panels A and B depict intensity histograms extracted from digital images obtained with Aperio and Leica respectively. Color lines indicate intensities in the red, green and blue channels as well as their grayscale transformation (black line). Inserts provide examples of slide contents from breast cancer samples stained with the HER2 antibody. Discrepancies in coloration in the immunohistochemistry images correspond to the differences in histogram shapes of all three channels. C) Relationships between white pixels acquired by Aperio and Leica slide scanners for a slide stained with HER2 antibody. The membrane staining was evaluated in the range of white pixels from 120 to 240 (See methods section). D) Same as C) but for a slide stained with ER antibody. A linear regression model was used to fit the data.

Gertych et al.

**Supplementary Figure 2**


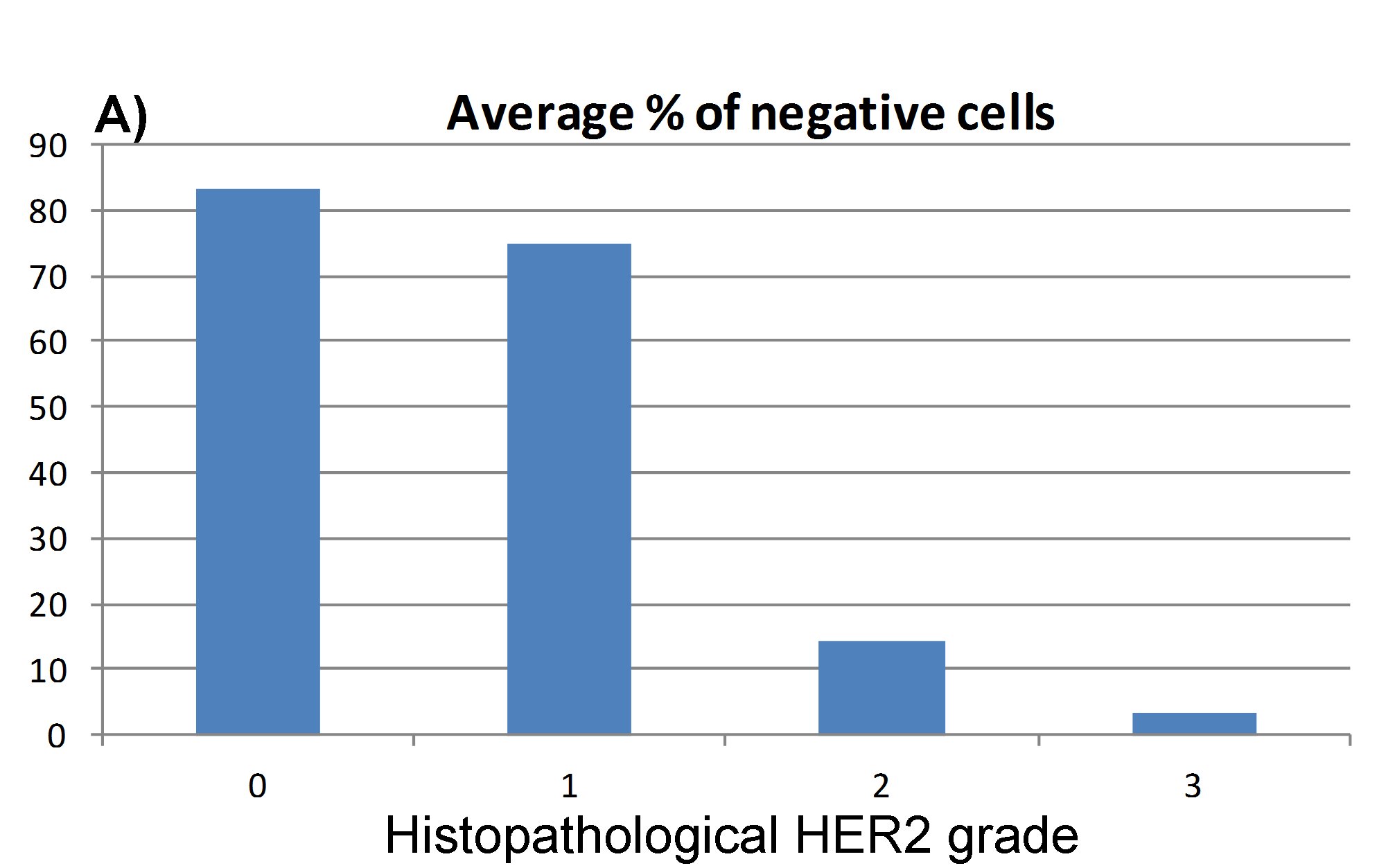

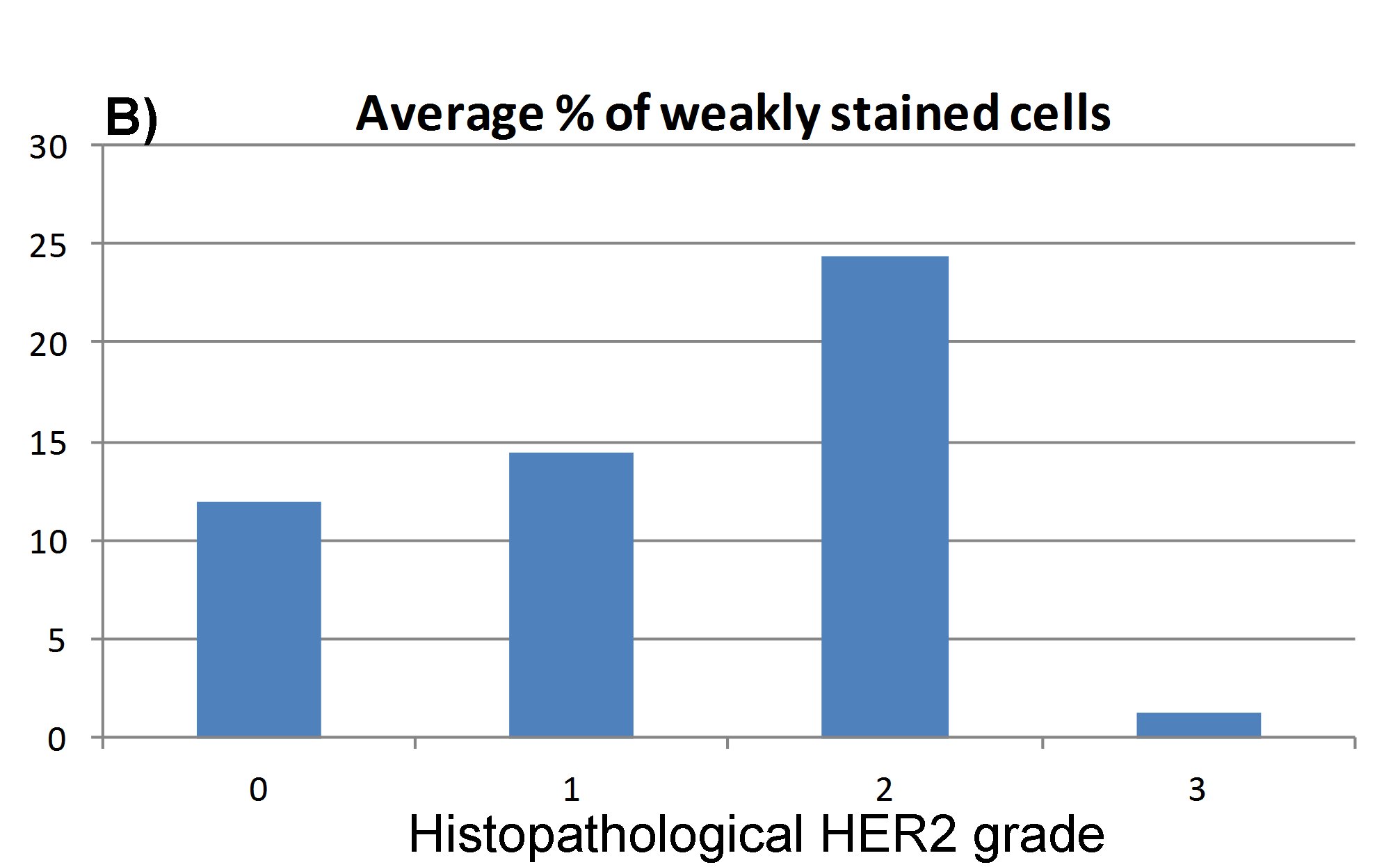

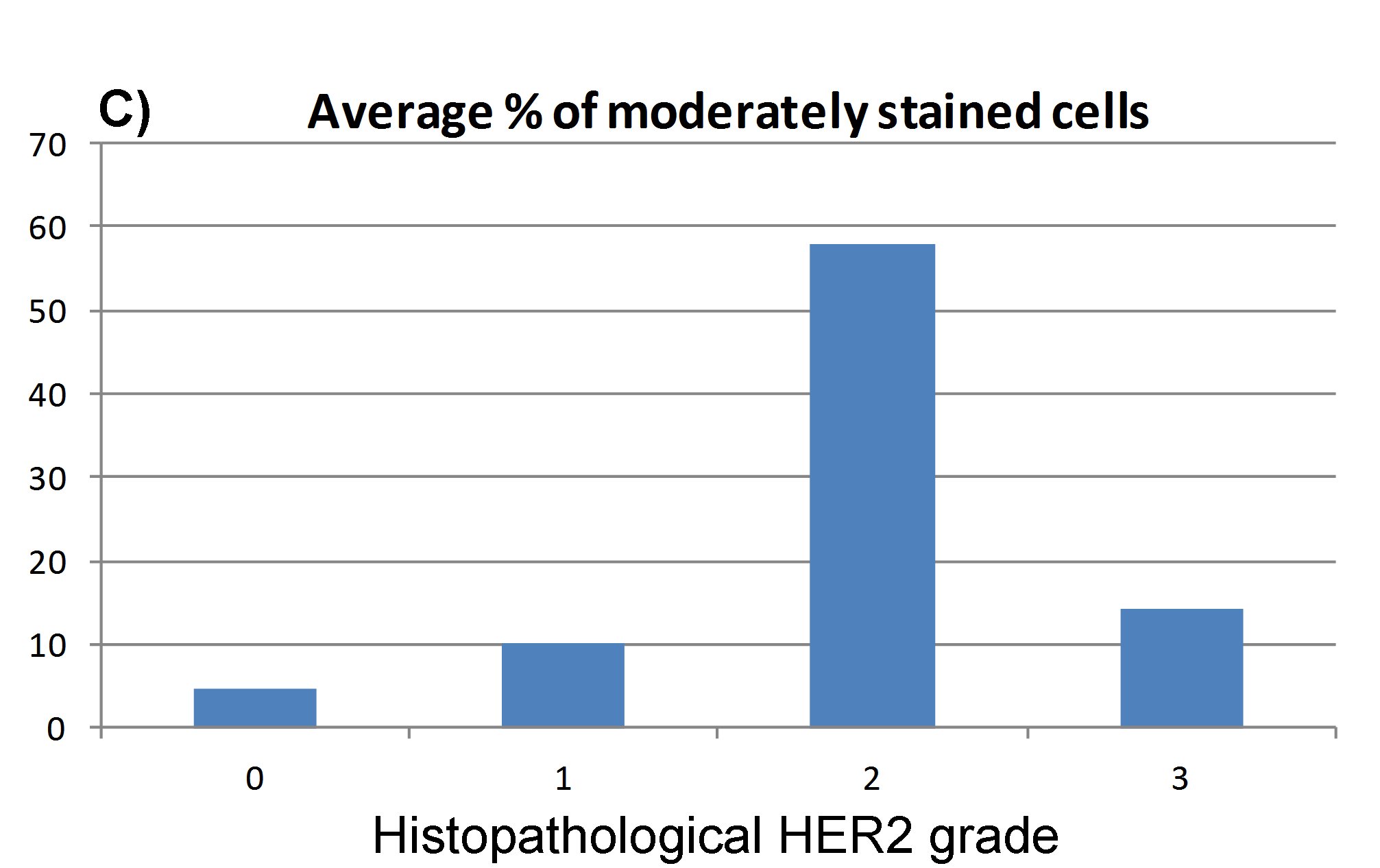

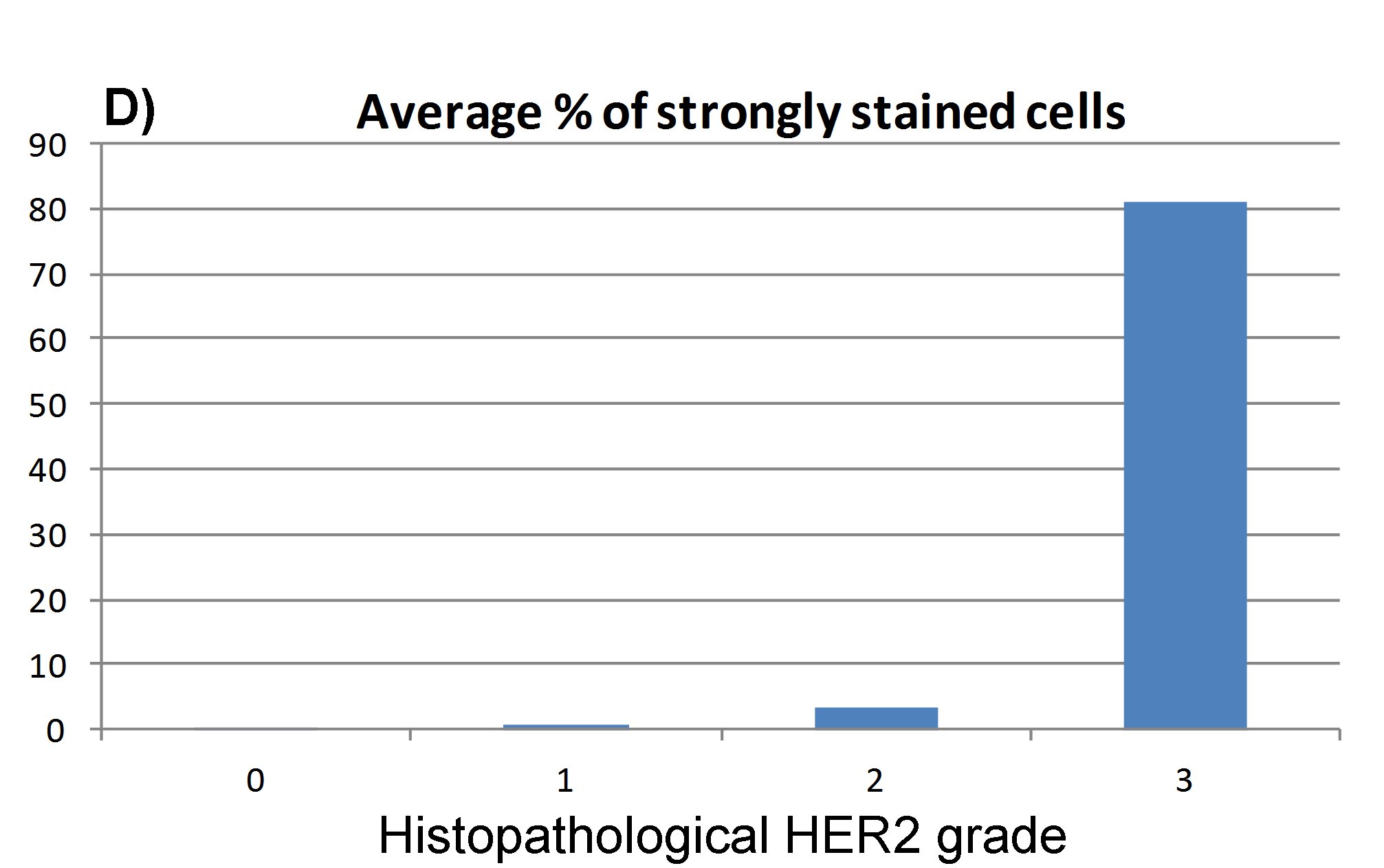


**Supplementary Figure 2.** **Distribution of cells from all slides within three categories of HER2 staining intensity**. Three staining categories were defined based on thresholds (t1 and t2) shown in Figure 1. Each slide received a pathological score of HER2 expression of 0, 1, 2 or 3. HER2 staining was quantified in five regions and the staining score was assigned to one of the three staining categories. Panel A shows the distribution of cells with negative or weak positivity in slides that were graded between 0 and 3 by a pathologist. Panel B, and C show the distribution of cells with moderate and strong positivity respectively. In total 87 regions from 15 slides were analyzed.
